# COST action TD1407: network on technology-critical elements (NOTICE)—from environmental processes to human health threats

**DOI:** 10.1007/s11356-015-5221-0

**Published:** 2015-08-20

**Authors:** A. Cobelo-García, M. Filella, P. Croot, C. Frazzoli, G. Du Laing, N. Ospina-Alvarez, S. Rauch, P. Salaun, J. Schäfer, S. Zimmermann

**Affiliations:** Instituto de Investigacións Mariñas (IIM-CSIC), 36208 Vigo, Spain; Institute F.-A. Forel, University of Geneva, Route de Suisse 10, CH-1290 Versoix, Switzerland; Earth and Ocean Sciences, School of Natural Sciences, National University of Ireland, Galway (NUIG), Galway, Ireland; Istituto Superiore di Sanità, via Giano della Bella 34, 00162 Rome, Italy; Laboratory of Analytical Chemistry and Applied Ecochemistry, Ghent University, Coupure links 653, B-9000 Ghent, Belgium; Applied Analytical Chemistry Laboratory, University of Warsaw, 02-093 Warsaw, Poland; Department of Civil and Environmental Engineering, Chalmers University of Technology, 41296 Gothenburg, Sweden; School of Environmental Sciences, University of Liverpool, 4, Brownlow Street, Liverpool, L693GP UK; University of Bordeaux, UMR EPOC 5805, Allée Geoffroy St Hilaire, 33615 Pessac, France; Aquatic Ecology and Centre for Water and Environmental Research, University of Duisburg-Essen, D-45117 Essen, Germany

**Keywords:** Technology-critical elements, COST Action, Analytical determination, Environmental biogeochemistry, Human exposure, (Eco)-Toxicology

## Abstract

The current socio-economic, environmental and public health challenges that countries are facing clearly need common-defined strategies to inform and support our transition to a sustainable economy. Here, the technology-critical elements (which includes Ga, Ge, In, Te, Nb, Ta, Tl, the Platinum Group Elements and most of the rare-earth elements) are of great relevance in the development of emerging key technologies—including renewable energy, energy efficiency, electronics or the aerospace industry. In this context, the increasing use of technology-critical elements (TCEs) and associated environmental impacts (from mining to end-of-life waste products) is not restricted to a national level but covers most likely a global scale. Accordingly, the European COST Action TD1407: Network on Technology-Critical Elements (NOTICE)—from environmental processes to human health threats, has an overall objective for creating a network of scientists and practitioners interested in TCEs, from the evaluation of their environmental processes to understanding potential human health threats, with the aim of defining the current state of knowledge and gaps, proposing priority research lines/activities and acting as a platform for new collaborations and joint research projects. The Action is focused on three major scientific areas: (i) analytical chemistry, (ii) environmental biogeochemistry and (iii) human exposure and (eco)-toxicology.

## Background

Approximately 99.7 % of the upper continental crust is composed of a relatively small number of elements (Si, Ti, Al, Fe, Mn, Mg, Ca, Na, K, P), whereas the vast majority of the naturally occurring chemical elements—the so-called ‘minor’ or ‘trace’ elements—account only for the remaining 0.3 % (Rudnick and Gao 2003). The concentrations of these trace elements in the Earth span over several orders of magnitude, from several hundreds of microgrammes per gramme down to tens of picogrammes per gramme. Despite their low concentrations, the discovery and use of several trace elements by humans can be traced back several thousands of years; Au (6000 BC), Cu (4200 BC), Ag (4000 BC) or Pb (3500 BC), amongst others. The massive requirements of these trace elements for a variety of technological applications, especially after the industrial revolution in the late eighteenth century, led to their extensive extraction from the lithosphere and resulted in the worldwide dispersion and remobilization of these elements within the biosphere. The development of new analytical technologies during the past decades enabled the determination of their speciation and concentration in a wide range of environmental compartments and facilitated the study of their environmental cycling and fate (e.g. Salbu and Steinnes 1995). The deleterious effects of some of these elements to living organisms have been well documented (Fairbrother et al. 2007) and have underpinned the development of a range of environmental guidelines, policies and laws (e.g. EU Water Framework Directive; WHO Drinking Water Guidelines). These were put into place to control the adverse effects of such elements (e.g. As, Cd, Cr, Cu, Hg, Pb) in their various chemical forms/species.

However, whilst considerable progress has been made in understanding the environmental fate and eco-toxicological behaviour of the more traditionally used elements mentioned above, the use of a further range of trace elements (whose inherent properties are required for use in an ever expanding list of new technologies) is rapidly increasing (Karn 2011). These of elements which includes Ga, Ge, In, Te, Nb, Ta, Tl, the platinum group elements (PGEs: Pt, Pd, Rh, Os, Ir, Ru)—Table 1—and most of the rare earth elements (REEs: Y, La, Ce, Pr, Nd, Sm, Eu, Gd, Tb, Dy, Ho, Er, Yb, Lu)—Table 2—are now essential components in a variety of applications including information and telecommunications technology, semiconductors, electronic displays, optic/photonic or energy-related technologies (Eggert 2011; Karn 2011; (APS and MRS 2011). Their current importance is such that several of these elements have now been labelled as ‘energy-critical elements’ or ‘technology-critical elements’ (TCEs; Table 1), and initiatives at national levels are underway to secure their availability in the coming years (APS and MRS 2011).

**Table 1:** Applications of technology-critical elements, except REEs

Element	Compounds	Applications
Gallium	GaAs, GaN	**Wafers** for (i) **Integrated circuits** in defence applications, high-performance computers, and telecommunications equipment. (ii) Optoelectronic devices (LEDs, photodetectors, solar cells) in aerospace applications, consumer goods, industrial, medical, and telecommunications equipment
	Trimethyl Ga, triethyl Ga	Epitaxial layering process for the production of LEDs
	CuNbGaSe (CIGS)	Thin film solar cells
Germanium	Ge oxide	**Fibre optic** systems in telecommunications, wide-camera lenses, microscopy
	Ge	**Infrared optics** in thermal imaging cameras, night-vision devices, spectroscopes
	Ge	Substrate for wafers for high-efficiency photovoltaic cells
	Ge oxide	Polymerization catalysts in PET production
	Ge single crystals	Semiconductor detectors (airport security), monochromators for beamlines in synchrotron X-ray diffraction
Indium	Indium tin oxide (ITO)	**Transparent conductive thin-film coatings** on flat-panel displays (e.g., LCDs)
	Indium metal	Solders and low melting point metal alloys (e.g., dental alloys, white gold alloys, substitute for Hg, nuclear control rods)
	InSb, InAs, InP, InGaAs	III–V semiconductor materials for LED and laser diodes in data transmission and displays, fibre optic communications
	CuNbGaSe (CIGS)	Thin-film solar cells
Niobium	HSLA ferro-Nb (60 % Nb), Nb metal	**High-grade structural steel** for vehicle bodies, oil and gas pipelines, architectural steels
	Nb oxide	Ceramic capacitors, glass coatings, camera lenses
	Vacuum-grade ferro-Nb, NiNb	Superalloys for jet engines and turbine blades
	Nb carbide	Cutting tools, railway tracks, ship hulls
	Nb powder, Nb oxide, Li niobate	Capacitors, surface acoustic wave filters (sensor and touch screen technologies)
	Nb_3_Ge, Nb_3_Sn, SbTi	Superconducting magnets in MRI scanners, NMR, particle accelerators
PGEs	Pd, Pt, Rh metals	**Catalytic converters** for the car industry
	Pt metal	Catalyst in fertliser and explosive industries, fabrication of speciality silicones, refining of petroleum, magnetic coating of computer hard discs
	Pt compounds (cisplatin, carboplatin, …)	Chemotherapy
	Pd metal	Multi-layer ceramic capacitors, hybrid integrated circuits, platting of connectors in computers
	Pd, Pt, Rh alloys	Vessels for molten glass
	Pd, Pt metals and alloys	Dental restorative materials
	Pt, Pd metals	Jewellery
	Pt, Pd, Rh metals	Exchange traded products as investment
	Ru	Electronics (chip resistors, flat screen plasma displays and hard discs)
	Ir	Crucibles for the electronics industry
	Os alloys	High wear applications such as instrument pivots and electrical contacts
	Os tetroxide	Fatty tissue staining for optical and electron microscopy
Tantalum	Ta oxide	**Capacitors** in automotive electronics, personal computers, mobile telephones
	Ta oxide	Glass coatings, camera lenses, catalyst
	Ta carbides	Cutting tools
	Ta halides	Catalysts in the (petro)chemical industry
	Ta metal	Pacemakers, prosthetic devices
Tellurium	CdTe	**Solar cells**
	HgCdTe, BiTe	**Thermal imaging** for infrared sensors, **thermocooling** devices, and electronics and consumer products
	Te metal	Alloying additive in steel, Cu alloys, Pb alloys, cast Fe
		Vulcanizing agent and accelerator in rubber production Catalyst in synthetic fibre production Pigment in glass and ceramics
Thallium	Tl-metal	**Contrast agent** in noninvasive clinical screening (**scintigraphy**)
	Tl_2_S, SeTl_2_, TBCCO	High-temperature **superconductor** (HTS) and thermoelectric material (microstrip bandpass filters for wireless communications)
	Tl_3_ AsS_3,_, TI_3_PSe_4_	**Crystal filters** for light diffraction in acousto-optical measuring devices
	Tl(III) salts	Oxidation of olefinic compounds
	Thallium(I) iodide	Component in arc tubes of metal-halide lamps, to increase the light intensity
	TlBr-TlI (KRs-5)	Attenuator for reflection of prisms in IR spectroscopy

Main uses in bold. Source: http://minerals.usgs.gov/minerals/pubs/commodity ; http://www.bgs.ac.uk/mineralsuk/home.html

*ITO* indium tin oxide, *HSLA* high-grade structural steel, *LCDs* liquid crystal displays, *LEDs* light-emitting diodes, *MRI* magnetic resonance imaging, *NMR* nuclear magnetic resonance, *PET* polyethylene terephthalate, *PGEs* platinum group elements (Pt, Pd, Rh, Ir, Ru, Os), *TBCCO* thallium-barium-calcium-copper oxide

**Table 2:** Main uses of rare earth elements (Y, La, Ce, Pr, Nd, Sm, Eu, Gd, Tb, Dy, Ho, Er, Yb, Lu). Source: British Geological Survey (http://www.bgs.ac.uk/mineralsuk/home.html)

Applications	Elements
Catalysts Petroleum refining Catalytic converter Diesel additives Chemical processing Industrial pollution scrubber	La, Ce (Pr, Nd)
Ceramics Capacitors Sensors Colourants Scintillators Refractories	La, Ce, Pr, Nd, Y, Eu, Gd, Lu, Dy
Glass and polishing Polishing compounds Decoulourisers/colourisers UV resistant glass X-ray imaging	Ce, La, Pr, Nd, Gd, Er, Ho
Magnets Motors Discs drives MRI Power generation Microphones and speakers Magnetic refrigeration	Nd, Pr (Tb, Dy)
Metallurgical alloys NimH batteries Fuel cells Steel Lighter flints Super alloys Al/Mg	La, Ce, Pr, Nd, Y
Phosphors Display phosphors CRT, LPD, LCD Fluorescent lighting Medical imaging Lasers Fibre optics	Eu, Y, Tb, Nd, Er, Gd (Ce, Pr)
Other	
Nuclear	Eu, Gd, Ce, Y, Sm, Er
Defence	Nd, Pr, Dy, Tb, Eu, Y, La, Lu, Sc, Sm
Pigments	Ce, Y

Due to their high economic relevance and the dependency of the European Union (EU) on imports, mainly from China, the EU has identified 14 critical materials (European Commission: Enterprise and Industry 2010) for which, at the moment, no mining zones with an acceptable short/mid-term profit exist within the EU borders. These critical 14 materials identified by the EU encompass most of the TCEs here defined, namely Ga, Ge, In, Nb, Ta, PGEs, and REEs.

## The need for an evaluation and assessment on the analytical, environmental and toxicological aspects of TCEs

The current significant gaps in our knowledge and understanding of TCEs, from their environmental levels and fate to their potential (eco)toxicological impact, are mainly explained by two factors: (i) their typical ultra-trace concentrations, making their analytical determination extremely difficult and/or time-consuming, and (ii) the absence of any significant industrial role (apart from some biomedical applications) prior to their current massive use following the increasing demand of new technological applications. However, this scenario is changing rapidly and substantially. The current use of TCEs in new technological products is inducing significant changes in the processes associated with their natural environmental cycle at the Earth’s surface. At all stages of their life cycle, these elements and their compounds can be released into the environment and come in contact with the biosphere. The wider impact of the increasing use of many TCEs within a range of environmental compartments is poorly understood; for several TCEs, there are basically no data at all.

As an example, anthropogenic disturbances in the geochemical cycles of the PGEs have recently been reported (Rauch et al. 2005; Cobelo-Garcia et al. 2013); accordingly, elevated and rapidly increasing PGEs concentrations have been measured at urban sites in Western Europe (Schäfer et al. 1999; Cobelo-García et al. 2011), the USA (Rauch et al. 2004) and an increasing number of countries worldwide (e.g. in Ghana: Kylander et al. 2003; in Mexico: Rauch et al. 2006), as well as at remote sites (Rauch et al. 2005); also, preliminary data demonstrate the contamination of the food chain (Frazzoli et al. 2007). Although there is evidence for anthropogenic PGEs nanoparticles cycling between different environmental compartments (road sites-aquatic systems, soil, etc.), including plant uptake (Schäfer et al. 1998), there is to date no systematic follow-up on pathways and transfer mechanisms. Also, the disturbance of the natural environmental distributions of several rare-earth elements (REEs) has been recently reported in waters of the Rhine River, Germany (e.g. Kulaksiz and Bau 2013), and San Francisco Bay, USA (Hatje et al. 2014), indicating that human activities are already impacting the geochemical cycles of these elements. For many elements, however, even the current concentrations in environmental systems are unknown (Filella et al. 2014; Biver et al. 2015). In general, the current information is insufficient to support even the calculation of mass balances, sources and/or sinks for TCEs on a global or even regional (e.g. Europe) scale. Of further concern is that, despite their widespread use, current knowledge does not support the application of robust risk assessment processes and, as a consequence, they are not included in regulations (in contrast to those available for other metals with a longer record of use).

## Action’s areas of interest and objectives

It is within this context that it is timely and relevant to push forward a coordinated scientific effort to improve our basic understanding of the behaviour of the TCEs. This needs a widespread approach to the TCEs, from the processes underpinning their environmental behaviour, the potential threat to human health and what is required in terms of monitoring, assessment and regulation, as well as raising public awareness and providing critical information to inform debate on the issues surrounding TCEs. In this context, the overall objective of this COST action network is defined thus: The creation of a network of scientists and practitioners interested in TCEs, from evaluating their environmental processes to understanding potential human health threats, with the aim of defining the current state of knowledge and gaps, proposing priority research lines/activities and acting as a platform for new collaborations and joint research projects. The Action is therefore focused on three major scientific areas: (i) analytical chemistry, (ii) environmental biogeochemistry and (iii) human exposure and toxicology.

## Action’s organization

The Action is chaired by A. Cobelo-García from the Spanish National Research Council (CSIC) at the Instituto de Investigacións Mariñas (IIM) and vice-chaired by M. Filella from the University of Geneva (Switzerland) at the Institute F.-A. Forel. In order to attain its objectives, the Action is organized in four working groups (WG) with the following tasks:

1. Analysis and intercalibration (Leader: P. Croot; Co-leader: P. Salaun)
  This WG will (i) evaluate the most appropriate currently available procedures for the determination of TCEs and their chemical and physical species, especially those with known or suspected deleterious effects, in environmental and biological samples; (ii) propose directions for the development of new analytical strategies with the aim of decreasing the analysis time and costs, allowing routine monitoring; (iii) promote community-wide inter-laboratory exercises to ensure analytical accuracy and (iv) seek interaction with relevant institutions for the development of appropriate certified reference materials for TCEs. An expected product of WG1 is an analytical ‘cookbook’ providing details and best practice advice on suggested protocols for accurate measurement of selected TCEs.
2. Environmental impact and cycling (Leader: S. Rauch; Co-leader: J. Schäfer)
  The WG will address different aspects of the environmental fate of TCEs. It will (i) condense the available information on environmental TCEs concentrations from national and international databases across Europe; (ii) complement the existing data by performing measurements and incubation experiments to create more complete pictures on the human impact on the TCEs cycles and (iii) interact with, and complement, existing efforts on TCEs’ sustainable exploration, extraction, processing and recycling in order to name sectors in which recycling and waste management strategies may be improved. The overall goal is to deliver new insights on the anthropogenically impacted biogeochemical TCEs cycle in Europe.
3. Human exposure and toxicology (Leader: C. Frazzoli; Co-leader: S. Zimmermann)
  This WG will critically collate the available data on (i) human exposure through direct (air inhalation, dermal absorption, soil ingestion) and indirect (diet) pathways; (ii) potential human health risks; (iii) (eco)toxicology; (iv) possible bioaccumulation and carry over in food chains and (v) mixture toxicology and bioavailability predictive modelling to assess possible markers of (early) exposure and effect in humans, animals and/or the environment, identifying the existing gaps that need to be addressed. Where existing data allows it, estimations of the human exposure to TCEs will be provided, indicating the main TCEs forms, species and mixtures of concern as well as the most sensitive and/or vulnerable groups of population, and identifying those cases where no information is currently available and are more critical for public health.
4. Training and capacity building (Leader: N. Ospina-Álvarez; Co-leader: G. Du Laing)
  Its remit will be to ensure that young researchers are adequately trained and that information about TCEs analytical determination, sustainable environmental resource management and exposure as well as (eco)toxicology and early markers is disseminated to where such expertise is lacking (e.g. authorities, industry and academic research).

## Planned activities

During the 4-year duration of the COST Action (01/06/2015 – 31/05/2019), several activities are planned in order to fulfil its objectives:

- Workshops: With the aim of bringing together scientists and stakeholders to present their research, discuss the current state of knowledge and identify the future research needs, two workshops are planned on (i) Environmental Concentrations, Cycling and Modeling of TCEs (late 2016), and (ii) Human Exposure and Toxicology of TCEs (mid-late 2017).
- Training Schools: Designed for graduate students and post-doctoral workers, and with the aim of introducing them in the latest advances, the Training Schools will feature lectures from leaders in the field and practical experience supervised by active practitioners. Two training schools will be held, covering (i) Analytical Protocols (from Sampling to Analysis) for TCEs, Measurement Uncertainty and Data Validation (mid-late 2016), and (ii) Methods for Impact Assessment (Environmental Cycling, Exposure and Toxicology) (late 2017–early 2018).
- Final Conference: Intended to attract a diverse audience, including analytical and environmental chemists, environmental modellers, resource managers, toxicologists, food safety assessors and managers, NGOs, industry and the media. Planned for the last year of the Action (early 2019).
- Short-Term Scientific Missions (STSMs): they will allow young researchers to make a stay at a research centre specializing in any particular area covered by the Action objectives. It is expected that up to 30 STSMs are completed during the life of the Action.

All the NOTICE COST Action objectives, activities, progress and results are available at the project website (www.costnotice.net) and disseminated via Twitter (https://twitter.com/NOTICE_COST).

## Perspectives

The current socio-economic, environmental and public health challenges that countries are facing clearly need common-defined strategies to inform and support our transition to a sustainable economy. Here, the technology-critical elements are of great relevance in the development of emerging key technologies—including renewable energy, energy efficiency, electronics or the aerospace industry. In this context, the increasing use of TCEs and associated environmental impacts (from mining to end-of-life waste products) is not restricted to a national level but covers most likely a global scale. Therefore, trans-national scientific coordinated activities—such as this European COST Action—are requested to tackle the proposed objectives through (i) optimization of human and material resources on TCEs research, (ii) training of researchers and capacity building, (iii) improving the quality and comparability of results and (iv) increasing the level of scale and scope of research activities on TCEs through the establishment of key research priorities. This will result in significant social benefits as the findings and conclusions derived from the Action’s activities will influence the decision-making bodies (e.g. through the identification of chemicals of concern, identification of contaminated sites, proposition of environmental quality guidelines, etc.) in order to enhance the environmental safety—including human exposure to these elements through food and water.
